# One and Then Another (Poetry)

**Published:** 1881-08

**Authors:** 


### ONE AND THEN ANOTHER.

One step and then another,
And the longest walk is ended;
One stitch and then another,
And the longest rent is mended;
One brick and then another,
And the highest wall is made;
One flake upon another,
And the deepest snow is laid.

So the little coral workers,
By their slow and constant motion,
Have built those pretty islands,
In the distant dark blue ocean;
And the noblest undertakings
Man's wisdom hath conceived,
By oft-repeated effort
Has been patiently achieved.

Then do not look disheartened
On the work you have to do,
And say that such a mighty task
You never can get through;
But just endeavor, day by day,
Another point to gain,
And soon the mountain which you feared
Will prove to be a plain.

Rome was not builded in a day
The ancient proverb teaches,
And nature, by her trees and flowers,
The same sweet sermon preaches.
Think not of far-off duties,
But of duties which are near,
And having once begun to work
Resolve to persevere.

